# Diet of otters (*Lutra lutra*) in various habitat types in the Pannonian biogeographical region compared to other regions of Europe

**DOI:** 10.7717/peerj.2266

**Published:** 2016-08-18

**Authors:** József Lanszki, István Lehoczky, Antoinette Kotze, Michael J. Somers

**Affiliations:** 1 Carnivore Ecology Research Group, Kaposvár UniversityKaposvár,Hungary; 2 Research Centre for Farm Animal Gene ConservationGödöllő,Hungary; 3 National Zoological Gardens of South AfricaPretoria,South Africa; 4 Genetics Department, University of the Free StateBloemfontein,South Africa; 5 Centre for Wildlife Management, Centre for Invasion Biology, University of PretoriaPretoria,South Africa

**Keywords:** Habitat type, *Lutra lutra*, Conservation, Food pattern, Non-native fish, Prey

## Abstract

Knowledge of the effect of habitat type and region on diet and feeding behaviours of a species facilitates a better understanding of factors impacting populations, which contributes to effective conservation management. Using spraint analysis and relative frequency of occurrence data from the literature, we described the dietary patterns of Eurasian otters (*Lutra lutra*) in 23 study sites within the Pannonian biogeographical region in Hungary. Our results indicated that diet composition varied by habitat type and is therefore context dependant. The differences among habitat types were however lower than expected. We noticed a decline in the fish consumption with a concomitant increase in trophic niche breadth and amphibian consumption in rivers, ponds (fish farms), backwaters, marshes and small watercourses. The main differences in diet were not attributed to the consumption of primary and secondary food types (fish and amphibians), but rather to differences in other, less important food types (mammals, birds). Using hierarchical cluster analysis, rivers and ponds could clearly be separated from other habitat types. We found the main fish diet of otters in most of these areas consisted of small (<100 g), eurytopic, littoral and non-native, mostly invasive species. Dietary studies from 91 sites in six European biogeographical regions showed that fish are consumed most frequently in the Atlantic and Boreal, less in the Continental and Pannonian, and least in the Alpine and Mediterranean regions. Comparative analysis indicated that the Mediterranean region (with frequent crayfish consumption) and Alpine region (frequent amphibian consumption) cluster separate from the other regions.

## Introduction

A thorough understanding of the diet, which largely defines the ecological niche of a species is an essential element in determining what limits populations (e.g., mortality, breeding success), and therefore essential for effective conservation management (Kruuk, 2006). For carnivores, food is often limited (Macdonald, 1983) and diets may vary between regions (e.g., Zalewski, 2004; Kruuk, 2006; Lozano, Moleon & Virgós, 2006). During these periods of food shortage less preferred foods are often taken. Understanding these limitations and differences allows for planning an effective context-specific conservation strategy. Furthermore, a theoretical basis for population density estimation and carrying capacity in different habitats is important for conservation management (Hayward, O’Brien & Kerley, 2007).

This study focuses on the Eurasian otter (*Lutra lutra* Linnaeus, 1758) which is widely distributed in Europe, Asia and part of North-Africa (Conroy & Chanin, 2002; EEA, 2009). In Europe the distribution range is from the Boreal, Atlantic, Alpine, to the Continental, Pannonian, Anatolian, Black Sea and Mediterranean biogeographical regions (Roekaerts, 2002). In Hungary, otters are widespread over 77.5% (72,000 km^2^) of the country, but are less common in the mountains and dry plain areas (Heltai et al., 2012). As otters sometimes cause fish stock losses in Hungary they are still persecuted (Lanszki, Orosz & Sugár, 2009), despite being legally protected.

The present study area (Hungary in the Pannonian biogeographical region) is surrounded by the Carpathian and Dinaric mountains and the Alps. These mountains have created a unique climatic region with a unique biodiversity (Varga, 1995). The climate is temperate with different climatic influences within the basin, i.e., Atlantic-alpine on the west, Mediterranean on the south and Continental in the east. The Carpathian basin is rich in surface waters but the rivers are mostly artificially regulated (Dövényi, 2010). Owing to differences in climate (and effects of soil, barriers, co-evolution, etc.), biodiversity in each biogeographical region differs (MacArthur, 1965).

The otter diet is well understood (Kruuk, 2006) making it an ideal species to compare diet across regions. The primary food of otters in Europe is fish (Jedrzejewska et al., 2001; Clavero, Prenda & Delibes, 2003; Kruuk, 2006; Krawczyk et al., 2016), with frogs or crayfish being of secondary importance. In comparing the diet of otters in Poland with those from Eurasia, Jedrzejewska et al. (2001) hypothesized that the type of habitat (coast, river, stream, lake) influences the diet. Similarly, Krawczyk et al. (2016) tested how fish prey of otters living in freshwaters depends on habitat type (vegetation, land use, water type, i.e. standing or flowing water). Other studies on diet composition include comparisons between countries (e.g., Ireland and Great Britain; McDonald, 2002), or within countries among some areas or habitat types (Kemenes & Nechay, 1990; Taastrom & Jacobsen, 1999; Ruiz-Olmo & Jiménez, 2009; Smiroldo et al., 2009). Within a single biogeographical region (under similar climatic conditions) little is known about how habitat type effects diet composition and feeding habits of otters.

Based on the hypothesis that otter diet composition is affected by habitat type on a continent or smaller scale, our first prediction is that trophic niche breadth and general dietary patterns of otters, on the basis of seven main food types, will vary by habitat type within the Pannonian biogeographical region. Each distinct study conducted in the Pannonian bioregion (Table S1) on lowland areas suggests that otters consume fish as primary food more frequently at ponds (fish farms) where fish availability (biomass) is high (Kemenes & Nechay, 1990; Kranz, 2000; Lanszki et al., 2001), than elsewhere. Other prey types had greater importance on near-natural or temporarily dry habitat types (Erlinge, 1967; Kemenes & Nechay, 1990; Clavero, Prenda & Delibes, 2003; Lanszki & Molnár, 2003; Lanszki & Sallai, 2006; Lanszki & Széles, 2006; Lanszki, Orosz & Sugár, 2009; Lanszki, Széles & Yoxon, 2009; Bauer-Haáz et al., 2014). With regard to the first prediction, we test MacArthur’s (1955) ecosystem stability hypothesis which states that dietary diversity is lower when the habitat is more stable, as has been found in otters previously (Jedrzejewska et al., 2001; Clavero, Prenda & Delibes, 2003; Ruiz-Olmo & Jiménez, 2009).

Focusing on the primary prey type (fish), our second prediction is that as fish consumption by otters is determined by characteristics of the fish (Table S1), due to differences in habitat characteristics, diet will vary by habitat type within the Pannonian region. As otters consumed mainly small fish in shallow waters (Erlinge, 1968; Carss, 1995; Kruuk, 2006), and their diet closely reflects the composition of local fish assemblages and densities (Erlinge, 1967; Kloskowski, 1999; Lanszki et al., 2001; Lanszki & Sallai, 2006; Bauer-Haáz et al., 2014), the composition of fish in the diet varies with habitat (Erlinge, 1967; Wise, Linn & Kennedy, 1981; Carss, Kruuk & Conroy, 1990; Kemenes & Nechay, 1990; Lanszki et al., 2001; Lanszki & Molnár, 2003; Kruuk, 2006; Lanszki & Sallai, 2006; Lanszki & Széles, 2006). In order to detect expected differences among feeding habits of otters living in different habitat types, we determine fish prey use by analysing the characteristics of consumed fish, such as size (weight) class, guild, habitat zone and origin of fish (i.e., native or alien). We test whether otters generally consume bigger, littoral and non-native fishes at ponds and lakes (Kemenes & Nechay, 1990; Lanszki, Orosz & Sugár, 2009; Bauer-Haáz et al., 2014), and whether they consume higher ratios of rheophilic fishes along flowing waters (Lanszki & Sallai, 2006; Lanszki, Széles & Yoxon, 2009), and native fish in natural or less modified habitats (Balestrieri et al., 2013; Bauer-Haáz et al., 2014), e.g., on rivers, backwaters and marshes, than elsewhere. Here we evaluate fish use without dietary preferences (Krebs, 1989).

Based on the hypothesis that biodiversity varies among biogeographical regions and dietary differences arising from geographical and ecological factors are experienced in larger scales, our third prediction is that there will be differences in the general diet composition of otters living in different biogeographical regions. In a European review, Clavero, Prenda & Delibes (2003) found differences in the diet of otters in the Mediterranean biogeographical region compared to that in the Temperate zone (all other bioregions other than the Mediterranean were in one group). In a Eurasian study, Jedrzejewska et al. (2001) found differences in diet compositions of otters among habitat types and also a strong relationship between trophic diversity and latitude. In Italy, Remonti, Balestrieri & Prigioni (2009) found a progressive reduction of fish consumption with stream elevation (and hence in smaller streams), with increasing amphibian (and locally crayfish) consumption. That is, besides habitat type, other habitat factors such as altitude (Remonti, Balestrieri & Prigioni, 2009), latitude (Jedrzejewska et al., 2001; Clavero, Prenda & Delibes, 2003), water level (Tumlison & Karnes, 1987), vegetation type and structure (Ruiz-Olmo & Jiménez, 2009; Krawczyk et al., 2016), along with abundance of fish and alternative prey types (Lanszki, Molnár & Molnár, 2006; Remonti, Balestrieri & Prigioni, 2009; Pagacz & Witczuk, 2010; Day, Westover & McMillan, 2015) may also influence otter diet. Following from these large scale differences in otter diet we expect that diet composition of otters varies, not only between the Mediterranean region and the Temperate zone (including the Pannonian one), but also among bioregions within the Temperate zone of Europe.

The aims of our study, based on known diet compositions of otter in the Pannonian biogeographical region were to (1) synthesize habitat specific differences in dietary patterns and trophic niche breadth, (2) assess habitat specific differences on the basis of fish prey characteristics (weight class, guild, habitat zone and origin of consumed fish), and (3) determine biogeographical region-dependent differences in otter diet.

## Materials and Methods

### Ethics statement

As we reviewed published otter diet papers, it did not involve any handling of animals, and no ethical clearance was required.

### Data management

We collected otter prey use data across Europe from the ISI Web of Science database up to 2013 and then reviewed and extracted information from 80 published studies that included 140 sites. To allow for comparison, and owing for variations in methods used, we included only studies based on spraint analysis from freshwater systems (Clavero, Prenda & Delibes, 2003; Krawczyk et al., 2016), excluding those using stomach analyses due to differential digestion of prey types (Putman, 1984). Furthermore we only used sources with results expressed as percentage relative frequency of occurrence (RFO, number of occurrences of a certain food type divided by total number of occurrences of all food types ×100) or those that contained N data (number of food items for the seven main food types) to calculate RFO. We discarded studies that only reported data as frequency of occurrence (FO), biomass estimates (BIO, estimated biomass ratio of main food types consumed), or volumetric (VOL) calculations. With the known limitations and advantages of occurrence data for diet studies (in general: Reynolds & Aebischer, 1991, otter: Carss & Parkinson, 1996; Jacobsen & Hansen, 1996; Lanszki et al., 2015), we used RFO data, because these (and/or N data) were reported in most of the studies. Large sample sizes are associated with lower rates of Type I error, and furthermore RFO values are considered to be highly suitable for inter-population comparisons in diet studies (McDonald, 2002; Clavero, Prenda & Delibes, 2003; Lozano, Moleon & Virgós, 2006; Zhou et al., 2010). Biomass estimates can be considered useful (Reynolds & Aebischer, 1991), and in some studies close correlations have been found between RFO and BIO data (e.g., Lanszki & Sallai, 2006; Lanszki, Széles & Yoxon, 2009).

Publications selected for numerical analyses met the following criteria: minimum sample size of 100 spraints, the data covered four seasons and the location and study area were well defined. Several studies reported otter diets from more than one habitat type or from different sites. We pooled results from different sites from the same region, and only included data from freshwater habitats (Clavero, Prenda & Delibes, 2003). Further, we only considered long term studies including all seasons. We separated the data into two periods, the winter-spring (cold) and the summer-autumn (warm) from the Pannonian studies. As less than half of the studies for the other biogeographical region described seasonal data, we did not perform seasonal analyses on those data. Study sites were classified into biogeographical regions assigned according to a biogeographical map developed under the Council Directive 92/43/CEE, and which formed the basis for this Pan-European extension (Roekaerts, 2002). For our analysis, we used 10 studies (23 sites) from Hungary (Pannonian biogeographical region, Table S1) and 39 studies (68 sites) from other European biogeographical regions (Table S2).

We distinguished seven main prey categories (types), namely: fish, amphibians, reptiles, birds, mammals, crayfish, and other aquatic invertebrates including molluscs. For studies performed in Hungary, we calculated the total number of prey species (or taxa; abbreviated as NPS) and the total number of fish families (abbreviated as NFF) present each study (Clavero, Prenda & Delibes, 2003). For all locations the trophic niche breadth was calculated using  Levins (1968) index: $B=1/\sum {p}_{i}^{2}$, where *p*_*i*_ is the relative frequency of the *i*th food item, and standardised across seven main food items: *B*_*A*_ = (*B* − 1)∕(*n* − 1), ranging between 0 and 1 (Krebs, 1989).

We identified five main freshwater habitat types: R, rivers; S, small watercourses (including streams and canals); B, backwaters (or oxbow lakes); M, marshes (marshes, moors and bogs) and P, ponds (including fish ponds, water reservoirs and lakes). We categorized fish from studies in the Pannonian region based on four categories (see Lanszki et al., 2001; Lanszki & Sallai, 2006; Lanszki, Széles & Yoxon, 2009; Bauer-Haáz et al., 2014), i.e., (1) fish weight: <100 g, 101–500 g, 501–1,000 g and >1,000 g; (2) fish guild: R, rheophilic (preferring fast flowing water); E, eurytopic (tolerant of both flowing and standing waters); S, stagnophilic (preferring stagnant waters); (3) main habitat zone of fish: L, littoral (littoral, or shorezone), M, metaphyton (or aquatic plants, primarily shoreline reed grass, tangle, or under shoreline bush); P, pelagic (occurring in open water areas); B, benthic (or occurring in the layer of water directly above the bed); and (4) fish origin: N, native; A, alien (non-native). The weight classes of preyed individual fish recorded in the studies were determined using a reference collection of bones from fish of known sizes (Lanszki et al., 2001). Detailed classifications on four fish characteristics are available in articles cited in Table S1 and listed in Appendix S1.

#### Variables selected and statistical analyses

We examined the feeding differences of otters among habitat types using an ANOVA (Bonferroni post hoc test) on the basis of seven main food categories, standardised trophic niche breadth (*B*_*A*_), and characteristics of fish consumed (weight, guild, habitat zone and origin categories). We arcsine transformed the RFO data before analyses. We compared winter-spring and summer-autumn standardised trophic niche breadth values within each habitat types using paired samples *t*-test. We used the Chi-square test for a distribution analysis of general diet composition (seven main prey categories), and fish diet composition of the otters (using weight class, guild, habitat zone and origin of fish) among habitat types. We evaluated the relationships between consumption of the seven main prey taxa, and between these and standardised trophic niche breadth (*B*_*A*_) using Pearson correlation (*r*_*P*_).

We used hierarchical cluster analysis (with between-groups linkage as cluster method and Euclidean distance as interval of measure, range from 0 to 100; e.g., McDonald, 2002) to compare diet composition (RFO) data recorded for different habitat types, periods and biogeographical regions. During the cluster analyses zero value occurred only once (from 100) for the Pannonian region and once (from 42) for the other regions.

We examined dietary differences among biogeographical regions and habitat types on the basis of main food categories using multivariate analysis of variance (MANOVA, Bonferroni post hoc test, GLM procedure in (SPSS, 1999) with type III sum of squares; dependent variable: RFO of each animal food type or *B*_*A*_; independent variables: biogeographical region and habitat type). When we evaluated the data from the different bioregions of Europe, a small number of studies on backwater (natural stagnant waters) habitats were combined with the studies on pond and lakes habitats. We accepted a minimum probability level of *P* < 0.05, except in the case of the four characteristics of fish consumed (Bonferroni correction, *P* < 0.0125).

## Results

### Pannonian biogeographical region

#### General diet composition

In the Pannonian data set the distribution of the seven main food types consumed by otters differed significantly between habitat types (Chi-square test: ${\chi }_{24}^{2}=722.05$, *P* < 0.0001). With the assessment of each food type separately (Table S1), no significant habitat specific differences were found in the consumption of fish as principal food type (ANOVA: *F*_4_ = 2.05, *P* = 0.130), or for amphibians, as a secondary food type (*F*_4_ = 1.25, *P* = 0.324). Significant differences were found only in the consumption of some less frequently eaten prey types. Otters living along rivers consumed more birds than those living on backwaters (*F*_4_ = 2.95, *P* = 0.049); along small watercourses they consumed more mammals than along backwaters (*F*_5_ = 3.33, *P* = 0.033); and otters living along small watercourses and backwaters consumed more aquatic invertebrates (without crayfish) than those living on rivers (*F*_5_ = 2.94, *P* = 0.049) (Table 1). With reptiles and crayfish, habitat specific differences were not significant (*F*_4_ = 0.45 − 0.61, *P* = 0.661–0.770).

**Table 1:** Diet composition (RFO, percentage relative frequency of occurrence) and standardized trophic niche breadth (*B*_*A*_) of otters in different habitat types in the Pannonian biogeographical region. Number of studies per habitat type is given in parentheses.

	Diet composition (RFO, mean ± SE)				

Food types	Rivers (3)	Streams (7)	Backwaters (3)	Marshes (4)	Ponds (6)
Fish	82.9 ± 2.53	60.2 ± 7.54	70.6 ± 2.61	70.1 ± 7.89	81.6 ± 3.83
Amphibians	5.1 ± 1.18	13.0 ± 3.23	12.8 ± 3.40	11.2 ± 3.06	7.7 ± 2.39
Reptiles	0.6 ± 0.07	1.1 ± 0.40	1.7 ± 1.04	0.7 ± 0.40	0.8 ± 0.36
Birds	6.7 ± 2.09	3.2 ± 0.61	1.1 ± 0.46	2.5 ± 1.13	2.6 ± 0.62
Mammals	1.0 ± 0.35	3.3 ± 0.53	1.1 ± 0.30	2.9 ± 1.31	1.3 ± 0.39
Crayfish	1.4 ± 0.90	7.0 ± 6.01	0.1 ± 0.10	2.9 ± 2.93	0.4 ± 0.26
Other invertebrates	2.3 ± 0.42	12.1 ± 3.01	12.6 ± 2.09	9.8 ± 2.93	5.8 ± 1.59
*B*_*A*_	0.07 ± 0.014	0.23 ± 0.053	0.15 ± 0.016	0.17 ± 0.067	0.08 ± 0.020

Significant negative associations were found between the consumption of fish as primary dietary item, and amphibians as secondary food items (Pearson correlation, *r*_*P*_ = − 0.809, *F*_1,21_ = 39.75, *P* < 0.001).

The mean standardised trophic niche breadth (*B*_*A*_) value in studies from the Pannonian region (Table S1) was 0.15. The difference between the highest and the lowest values (streams *vs*. rivers and ponds) was not significant (ANOVA, Bonferroni post hoc test: *F*_4_ = 2.09, *P* = 0.125, Table 1). A close negative relationship was found between fish consumption and standardised trophic niche-breadth (Pearson correlation, *r*_*P*_ = − 0.94, *P* < 0.001), and *B*_*A*_ values increased with increasing consumption of amphibians (*r*_*P*_ = 0.74, *P* < 0.001), reptiles (*r*_*P*_ = 0.49, *P* = 0.005), mammals (*r*_*P*_ = 0.61, *P* < 0.001) or other aquatic invertebrates (not crayfish; *r*_*P*_ = 0.53, *P* = 0.002).

The mean number of fish families (NFF) in the diet of otters from the studies across the Pannonian region (Table S1) was 5.3 (range = 5.1–5.6), and the habitat-type difference in the total number of fish families was non-significant (ANOVA, Bonferroni post hoc test: *F*_4_ = 0.06, *P* = 0.993). The mean number of prey species (NPS) was 28.1, ranging from 23.0 (marshes) to 32.0 (rivers), with the habitat-type difference in number of prey species also non-significant (*F*_4_ = 1.44, *P* = 0.261).

**Figure 1:**
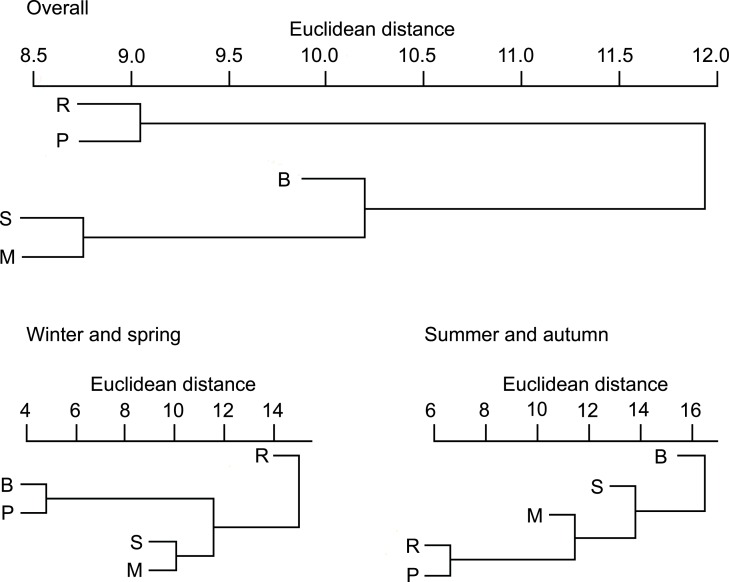
Similarity dendogram of the Euclidean distances between general diet compositions of otters from different habitat types of the Pannonian biogeographical region, overall, in winter-spring and summer-autumn periods. Habitat types: R, rivers; S, small watercourses; B, backwaters; M, marshes; P, ponds and lakes.

On the basis of Euclidean distances from the hierarchical cluster analysis, the dissimilarity in overall diet compositions of otters among habitat types was 15, and mean distance among all group pairs ranged between 9 and 23. Ponds (and lakes) and rivers where fish consumption was high (Table S1) fell into one group (Fig. 1). Other habitat types (small watercourses, marshes and backwaters), where consumption of amphibians was also in higher ratios fell into the second group.

**Figure 2:**
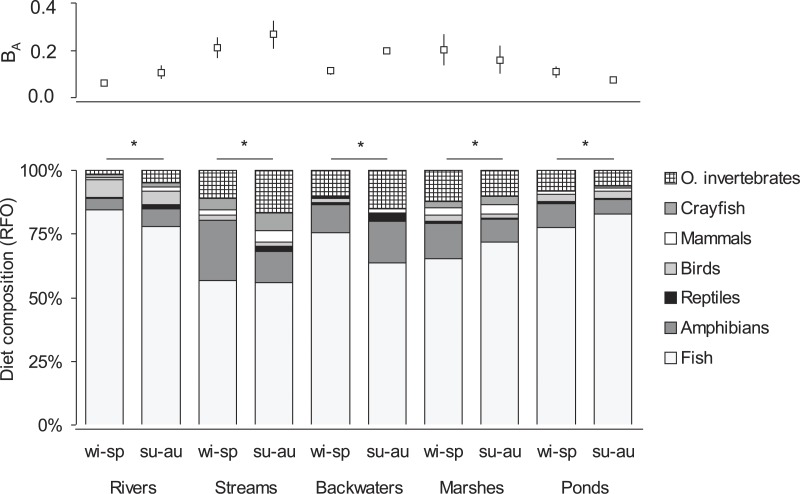
Seasonal diet composition (percentage relative frequency of occurrence) and standardised trophic niche breadth (*B*_*A*_, mean ± SE) of otters living in different habitat types of the Pannonian biogeographical region. ^∗^ denotes significant differences between winter-spring (wi-sp) and summer-autumn (su-au) periods within habitat types.

In the winter-spring period (Fig. 1), the diet composition (with considerable bird consumption beside fish consumption; Fig. 2) of otters along the rivers differed from the others (mean distance value: 21), with small watercourses and ponds falling into the second group (with least fish and most frequent amphibian consumption), and ponds and backwaters falling into the third group (with considerable amphibian consumption beside fish consumption). In the summer-autumn period (Fig. 1), backwaters and small watercourses (least fish and most frequent amphibian and other aquatic invertebrate consumption; Fig. 2) separated the most from marshes (with relatively frequent amphibian and other aquatic invertebrate consumption as well as frequent fish consumption) and from rivers and ponds (most frequent fish consumption). Within each habitat type, the winter-spring and the summer-autumn diet composition (Fig. 2) differed significantly (Chi-square test: ${\chi }_{6}^{2}=20.43$–142.45, *P* < 0.0001), while the differences between the seasonal standardised trophic niche-breadth values (Fig. 2) were not significant (paired samples *t*-test, *t*_2−6_ = 0.92–3.63, *P* = 0.069–0.408).

#### Fish prey characteristics

**Figure 3:**
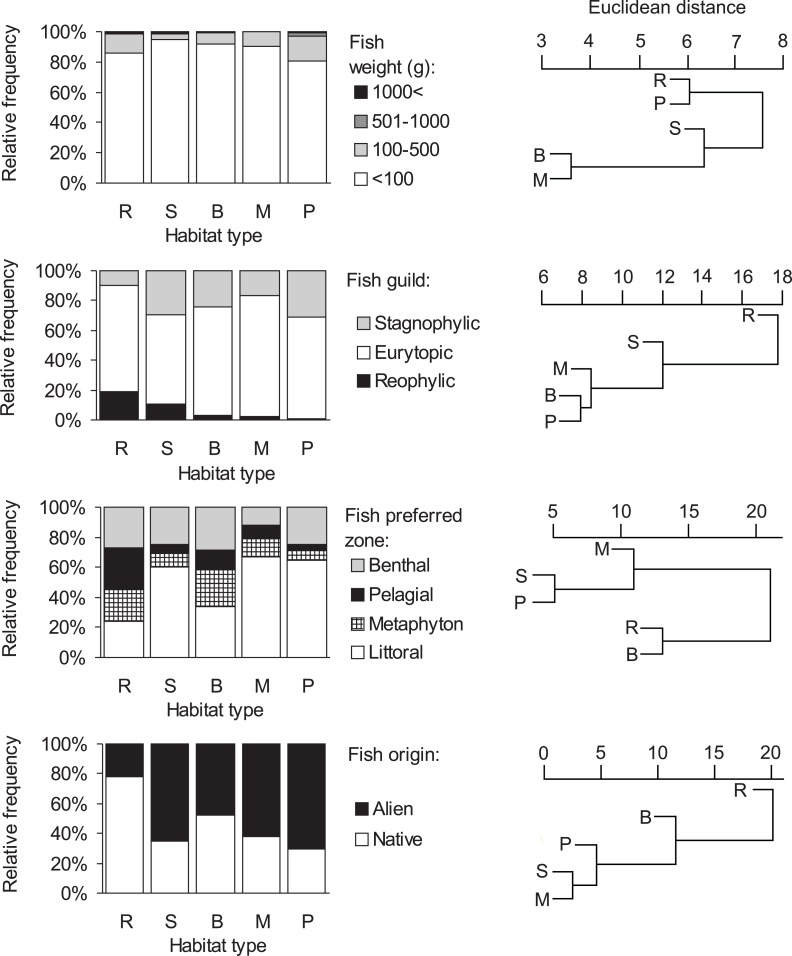
Distribution of fish types in the diet of otters and similarity dendogram of the Euclidean distances between fish food characteristics of otters living in different habitat types of the Pannonian biogeographical region. Habitat types: R, rivers; S, small watercourses, B, backwaters; M, marshes; P, ponds and lakes.

Consumption of small fish (<100 g) was predominant in all habitat types (>80%, Fig. 3, Table S1). The distribution of the four fish weight categories consumed by otters differed significantly between habitat types (Chi-square test: ${\chi }_{12}^{2}=910.89$, *P* < 0.001). Besides these biologically important differences, no statistically significant differences were found among habitat types within each (1-4) weight category (ANOVA, Bonferroni post hoc test: *F*_4_ = 1.46–2.21, *P* = 0.112–0.258). On the basis of hierarchical cluster analysis, the mean dissimilarity (Euclidean distance) between habitat types was 10 (range: 4–19). Ponds and rivers, where otters, besides eating small fish, consumed larger sized (>100 g) individuals relatively frequently (19.4 and 14.4%, resp.) fell into one group (Fig. 3), while other habitat types, where consumption of small fish was above 90% fell into the second group.

Consumption of eurytopic fish dominated in all habitat types (habitat type means, 60–81%, Fig. 3). Besides eurytopic fish, rheophilic fish were frequently eaten by otters on rivers (habitat type mean, 19%) and streams (11%), while, stagnophilic fish were frequently consumed in stagnant waters (17–31%) and streams (30%; Table S1). The distribution of the three fish guilds in the diet of otters differed significantly between habitat types (Chi-square test: ${\chi }_{8}^{2}=918.61,P\lt 0.0001$). A difference was found in the consumption of rheophilic fish (ANOVA, Bonferroni post hoc test: *F*_4_ = 3.40, *P* = 0.032) with otters living along rivers consuming more rheophilic fish than on ponds. For eurytopic and stagnophilic fish consumption, there were no significant differences among habitat types (*F*_4_ = 0.86–1.20, *P* = 0.346–0.509). The mean dissimilarity between habitat types was 16 (range: 8–27); with stagnant waters differing from running waters (Table S1, Fig. 3).

Littoral fish were predominantly eaten in all habitat types (habitat type means, 34–67%, Fig. 3), except for rivers (24%) where otters consumed in near equal frequency all four fish types (Table S1). The distribution of habitat zone categories of predated fish differed significantly between habitat types (Chi-square test: ${\chi }_{12}^{2}=1416.07,P\lt 0.001$), because otters living along rivers consumed more pelagic fish than in other habitat types (ANOVA, Bonferroni post hoc test: *F*_4_ = 3.47, *P* = 0.030). No habitat specific differences were found in the consumption of the other three (i.e., littoral, metaphyton and benthic fish) categories (*F*_4_ = 0.97–2.40, *P* = 0.084–0.451). The mean dissimilarity between habitat types was 21 (range: 5–30). Fish living in the littoral zone were eaten least frequently by otters along rivers and backwaters (first cluster in Fig. 3), while in other habitat types, where consumption of littoral fish was primary (>60%), it fell into the second cluster.

A significant difference (Chi-square test: ${\chi }_{4}^{2}=826.65$, *P* < 0.0001) was found in the distribution of fish origin (Fig. 3, Table S1) with habitat type. The difference was significant for both native and alien (or non-native fish) food types (ANOVA, Bonferroni post hoc test: *F*_4_ = 3.87, *P* = 0.021, for both). The mean dissimilarity between habitat types was 18 (range = 3–38). Native fish species were primarily consumed by otters living along rivers (78%; first cluster on Fig. 3), and in backwaters (approximately 50–50%; second cluster in Fig. 3), while in the rest of the habitat types alien fish species were mainly consumed (62–70%; third cluster in Fig. 3).

### Dietary patterns in different biogeographical regions

In the biogeographical regions studied, fish was the primary food type, followed by amphibians, except for the Mediterranean region, where crayfish was more important (Table 2). In the multivariate analysis of variance, bioregion dependent differences were found in the consumption of numerous food types (Table 2). Fish consumption of otters was more frequent in the Atlantic than in the Mediterranean region (MANOVA, Bonferroni post hoc test: *F*_5_ = 2.41, *P* = 0.045), reptile consumption was more frequent in the Mediterranean and Pannonian regions than in the other regions (*F*_5_ = 14.44, *P* < 0.0001), bird consumption was more frequent in the Continental, Pannonian, Boreal and Atlantic regions than in the Alpine and Mediterranean regions (*F*_5_ = 5.92, *P* < 0.0001), crayfish consumption was more frequent in the Mediterranean than in the Pannonian and Atlantic regions (*F*_5_ = 4.22, *P* < 0.002), and the consumption of other aquatic invertebrates was more frequent in the Alpine, Pannonian and Mediterranean regions than in other regions (*F*_5_ = 3.26, *P* = 0.010). No significant bioregion-dependent differences were found in the consumption of amphibians (*F*_5_ = 0.90, *P* = 0.487) and mammals (*F*_5_ = 1.25, *P* = 0.297).

**Table 2:** Diet composition (percentage relative frequency of occurrence) and standardized trophic niche breadth (*B*_*A*_) of otters in different biogeographical regions. Number of studies per habitat type is given in parentheses.

	Diet composition (RFO, mean ± SE)						

Food types	Pannonian (23)	Boreal (6)	Atlantic (19)	Continental (23)	Alpine (5)	Mediterranean (15)	Average
Fish	71.8 ± 3.31	78.5 ± 5.91	77.8 ± 3.23	75.9 ± 3.15	64.4 ± 5.49	62.1 ± 4.66	72.5 ± 1.69
Amphibians	10.2 ± 1.40	8.4 ± 2.67	10.1 ± 1.89	9.7 ± 2.69	22.4 ± 4.78	9.9 ± 2.65	10.6 ± 1.04
Reptiles	1.0 ± 0.21	0.0	0.01 ± 0.01	0.04 ± 0.03	0.04 ± 0.04	1.6 ± 0.39	0.5 ± 0.10
Birds	3.1 ± 0.49	3.1 ± 0.70	2.8 ± 0.48	4.2 ± 0.91	0.2 ± 0.20	1.0 ± 0.36	2.8 ± 0.31
Mammals	2.1 ± 0.35	7.0 ± 2.98	2.8 ± 0.86	1.2 ± 0.42	1.6 ± 0.73	2.3 ± 1.15	2.3 ± 0.37
Crayfish	2.9 ± 1.89	1.3 ± 0.46	2.3 ± 1.65	5.9 ± 1.57	1.8 ± 0.94	14.4 ± 3.59	5.2 ± 1.01
Other invertebrates	8.8 ± 1.32	1.8 ± 0.36	4.2 ± 1.71	3.2 ± 0.61	9.5 ± 5.65	8.7 ± 2.13	6.0 ± 0.73
*B*_*A*_	0.15 ± 0.023	0.11 ± 0.038	0.11 ± 0.020	0.11 ± 0.018	0.18 ± 0.038	0.21 ± 0.034	0.14 ± 0.011

Otters living along small watercourses consumed significantly (*F*_3_ = 3.54, *P* < 0.019) less fish (60.5%) than along rivers (73.4%) and ponds (or lakes; 78.8%), while marshes (76.9%) did not differ significantly from these habitat types. With other food types the habitat specific differences were not significant (*F*_3_ = 0.32–1.23, *P* = 0.306–0.808). Bioregion × habitat type interactions were not significant in any of the cases (*F*_12_ = 0.41–1.50, *P* = 0.143–0.953).

**Figure 4:**
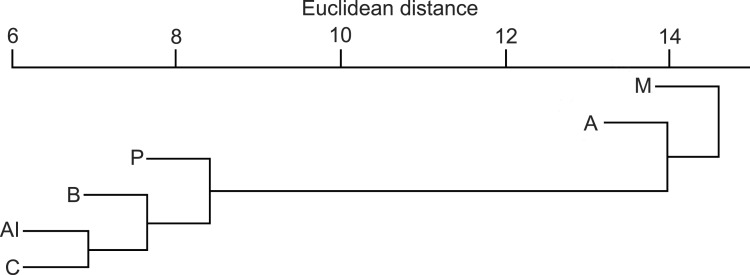
Similarity dendogram of the Euclidean distances between general diet compositions of otters from different European biogeographical regions. Biogeographical regions: B, Boreal; A, Atlantic; C, Continental; Al, Alpine; M, Mediterranean.

The Euclidean distances from the hierarchical cluster analysis (Fig. 4) showed that the mean dissimilarity in diet compositions of otters among biogeographical regions was 14 (range = 5–23). The Mediterranean region where fish consumption was the least and crayfish consumption the most frequent, and the Alpine region where fish consumption was the least and amphibian consumption was the most frequent, fell into two discrete groups, while other regions fell into the third cluster.

Trophic niche breadth (Table S2) was significantly higher in the Mediterranean region than in the Atlantic and Continental regions (MANOVA, Bonferroni post hoc test, *F*_5_ = 2.56, *P* = 0.035), and was significantly higher along small watercourses than on rivers and ponds (or lakes) (*F*_3_ = 3.99, *P* = 0.011). Bioregion × habitat type interaction was not significant (*F*_12_ = 0.98, *P* = 0.477).

## Discussion

### Habitat-specific differences in general dietary patterns and niche breadth

The diet of otters in the Pannonian biogeographical region varies among habitat types. Fish consumption decreased while amphibian consumption increased along a gradient from rivers, ponds, backwaters, marshes and small watercourses. However, Jedrzejewska et al. (2001) found that the fish component in the diet declines from 94% on sea shores to 71% on lakes and ponds, to 64% on rivers or streams, while the importance of amphibians and crayfish increases. Even so, data used in this analysis originated from different sample types and calculation methods, and several Eurasian areas. Our first prediction was only partially supported, because within the Pannonian region the main differences did not originate from consumption of primary and secondary foods (fish and amphibians), but rather from differences in other less important food types. Small watercourses that have a high risk of drying out (Lanszki, Széles & Yoxon, 2009) are of interest as this may affect the prey use of otters (Lanszki, Molnár & Molnár, 2006; Lanszki & Széles, 2006; Lanszki, Széles & Yoxon, 2009). In these habitats, fish consumption of otters did not differ statistically from areas that have bigger and more stable fish resources (ponds, backwaters and rivers; (Lanszki et al., 2001; Lanszki & Sallai, 2006)); this may however be due to a high variance in the data.

Otters in the Pannonian region rarely preyed on non-fish food types (Table S1). The balanced number of fish families and non-significant differences among number of prey species consumed (Table S1) confirmed a small difference in their diet composition. This corresponds to the cluster analysis; rivers and ponds where the fish consumption was the highest separated from the other habitat types, but the differences among habitats (overall and seasonally) were low. These results contradict other findings (Jedrzejewska et al., 2001), where differences among habitat types were considerable.

Despite that remarkable biological differences were found in the standardised trophic niche breadth values (similar to fish and amphibian consumptions), differences among habitat types and seasons were not statistically significant. This is not what we expected following the ecosystem stability hypothesis (MacArthur, 1955) (see also: Clavero, Prenda & Delibes, 2003; Ruiz-Olmo & Jiménez, 2009) which would suggest that we should find significant differences. However, *B*_*A*_ values on small watercourses were three times higher than on rivers and ponds. These results did not support our first prediction. The lack of significant differences is probably owing to the low mean in every case (0.07–0.23) and large SE values.

### Habitat-specific differences in fish prey characteristics

In general, the dietary composition of fish in otters corresponds to the local fish communities (Erlinge, 1968; Lanszki et al., 2001; Lanszki & Sallai, 2006; Bauer-Haáz et al., 2014), but we also found habitat-specific differences in the fish content, which corroborated our second prediction. For example, differences among habitat types on the basis of fish weight distributions in otter diets were low. These data from the Pannonian region (Table S1) supports those studies (Erlinge, 1969; Wise, Linn & Kennedy, 1981; Kruuk & Moorhouse, 1991; Kloskowski, 1999; Taastrom & Jacobsen, 1999; Copp & Roche, 2003) where it was found that otters mainly consume small fish. Preference calculations (Lanszki et al., 2001; Lanszki et al., 2007) indicated that under particular circumstances (e.g., in wintering fishponds where high numbers of large sized fish are available in a relatively small water body) otters prefer bigger fish. Predation upon larger-sized fish depends on their availability and the metabolic benefit to the otter (Erlinge, 1967; Carss, 1995; Kruuk, 2006). Small differences derived by hierarchical cluster analysis of habitat types support the importance of small (<100 g) fish for otters.

As expected, consumption of rheophilic fish was far more frequent on rivers and small watercourses than on ponds. However, contrary to what we expected, the consumption of stagnophilic fish species on small watercourses was similar to that on fishponds. In general, consumption of eurytopic fish species dominated in every habitat type. Higher fish content in spraints from flowing water habitats demonstrated that otters ate fewer of the faster rheophilic, and more of the slower stagnophilic and eurytopic fish (Lanszki & Sallai, 2006) here. This probably influenced the lower consumption of rheophilic fish on flowing waters. Periodical drying up or low water levels of regulated small watercourses affects fish communities (Lanszki, Széles & Yoxon, 2009), and only fish species with wide ecological tolerances are able to subsist in these waters. Thus, the composition of dietary fish from spraint in stagnant water habitats was as expected. We assume that stagnophilic species from fishponds, along streams may reach the rivers (Lanszki & Sallai, 2006; Lanszki, Széles & Yoxon, 2009).

Data of fish consumption categorized by habitat zone correspond to observations (Erlinge, 1968; Mason & Macdonald, 1986; Kruuk, 2006) where otters prey mainly in littoral zones, and less on benthic and pelagic zones. Similarly, rivers differed from the general pattern, because here, consumption of fish from each habitat zone was similar. However, small watercourses were similar to lakes, but separating habitat zones (mainly pelagic) on small watercourses was difficult.

The evaluation of the origin of consumed fish corresponds to the results of fish surveys and harvests in each area in the Pannonian region (fish farms: Lanszki et al., 2001; Lanszki et al., 2007; rivers and backwaters: Lanszki & Sallai, 2006; near-natural pond: Bauer-Haáz et al., 2014). These studies which determined preferences show that in both fish farms with high fish biomasses and abundances and in near natural areas with relatively lower fish abundances, otters prefer non-native fish or consume at around their occurrence in the environment. In a meta-analysis, Balestrieri et al. (2013) found an increased consumption of non-native fish species with time and towards south-western Europe. Our results highlight the extent of invasion of non-native fish species in Pannonian water courses which raises a key conservation concern for the different habitat types (Lanszki & Molnár, 2003; Lanszki & Sallai, 2006; Lanszki & Széles, 2006; Lanszki, Széles & Yoxon, 2009; Bauer-Haáz et al., 2014).

### Biogeographical region-dependent differences

Biogeographical region-dependent differences in consumption of numerous food types were noteworthy (Table 2), which supports our third prediction. Fish consumption decreased from the Atlantic and Boreal regions, the Continental and Pannonian to the Alpine and Mediterranean regions. This supports Clavero, Prenda & Delibes (2003) who found that the fish consumption was lowest in the South-European areas. In summary, the trophic niche breadth generally increases in a north-south direction. Clavero, Prenda & Delibes (2003) indicated a possible regional separation in the northern regions (northern from the Mediterranean one). Amphibian consumption was highest in the Alpine region, indicating that altitude is also an important factor (Remonti, Balestrieri & Prigioni, 2009), and ranged between 9–22%. Reptile consumption was highest in the Mediterranean and Pannonian regions while bird consumption decreased from the Continental region in Pannonian, Boreal, Atlantic to Alpine and Mediterranean regions. Mammal consumption was highest in the Boreal region, elsewhere it varied within from 1–3%. Consumption of crayfish decreased from the Mediterranean region to the lowest rate in the Pannonian, Boreal and Alpine region. Consumption of other aquatic invertebrates decreased from the Alpine, Pannonian and Mediterranean regions with the lowest consumption in the Boreal region.

The summarized data indicate that fish utilisation was habitat specific. Fish consumption decreased respectively in declining order in ponds or lakes, marshes, rivers and small watercourses. Considering the variety of food types in the diet, and their order of importance, the results partly correspond to those from Europe (Jedrzejewska et al., 2001; Krawczyk et al., 2016). The otter diet composition in the Pannonian region closely resembles the mean of all the studied regions. It indicates transient characteristics, with Continental, Mediterranean and Alpine properties influencing the climate, flora and the fauna of the Pannonian region (Varga, 1995). Unfortunately no data were available for the Steppic, Anatolian and Black Sea regions. Furthermore the low sample size from each region with high SE values may hide real differences. Although new scientific information over the last ten years on otter diet composition is available, our approach was more precautionary by applying more stringent comparisons (Jedrzejewska et al., 2001; Clavero, Prenda & Delibes, 2003).

*In conclusion*, we described and compared the dietary patterns of otters from different habitat types in the Pannonian biogeographical region, Hungary. We found habitat-specific differences in diet compositions, with relatively small variations among habitats when compared to other European biogeographical regions (Alpine, Atlantic, Boreal, Continental and Mediterranean).

##  Supplemental Information

10.7717/peerj.2266/supp-1 Table S1 Diet composition and fish distribution in otter (*Lutra lutra*) diets in the Pannonian biogeographical region (Hungary)

10.7717/peerj.2266/supp-2 Table S2 Diet composition of otters (*Lutra lutra*) in freshwater habitats of different biogeographical regions, in Europe

10.7717/peerj.2266/supp-3 Appendix S1 References for Tables S1 and S2

## Additional Information and Declarations

### Data Availability

The following information was supplied regarding data availability: The data can be found in the Supplemental Information.
