# Supplementary material for: Diet of otters (Lutra lutra) in various habitat types in the Pannonian biogeographical region compared to other regions of Europe
Source: PeerJ. 2016 Aug 18;4:e2266. doi: 10.7717/peerj.2266 (PMC4994076; doi:10.7717/peerj.2266)
Supplement: Table S1 [file peerj-04-2266-s001.doc]

**Table S1. Diet composition and fish distribution in otter (*Lutra lutra***) diets in the Pannonian biogeographical region (Hungary).

| Hab. | n | Food types | | | | | | | B_A_ | NPS | NFF | Fish weight | | | | Fish guild | | | Fish habitat zone | | | | Fish origin | | Source |
| --- | --- | --- | --- | --- | --- | --- | --- | --- | --- | --- | --- | --- | --- | --- | --- | --- | --- | --- | --- | --- | --- | --- | --- | --- | --- |
| type |  | F | A | R | B | M | C | I |  |  |  | 1 | 2 | 3 | 4 | R | E | S | L | M | P | B | N | A |  |
| R | 184 | 85.3 | 4.0 | 0.7 | 5.1 | 1.5 | 0.4 | 2.9 | 0.06 | 28 | 5 | 82.6 | 16.1 | 0.9 | 0.4 | 31.5 | 61.2 | 7.3 | 23.6 | 19.1 | 22.5 | 34.8 | 79.8 | 20.2 | 1 |
| R | 663 | 85.5 | 7.5 | 0.5 | 4.1 | 0.3 | 0.6 | 1.5 | 0.06 | 37 | 6 | 83.3 | 15.4 | 0.8 | 0.5 | 8.6 | 74.7 | 16.7 | 40.1 | 13.9 | 18.0 | 28.0 | 59.9 | 40.1 | 1 |
| R | 188 | 77.8 | 3.9 | 0.7 | 10.8 | 1.1 | 3.2 | 2.5 | 0.10 | 31 | 5 | 90.8 | 7.4 | 0.9 | 0.9 | 16.6 | 77.9 | 5.5 | 7.4 | 30.3 | 42.9 | 19.4 | 94.9 | 5.1 | 1 |
| S | 254 | 39.5 | 28.6 | 3.2 | 3.5 | 5.3 | 0.0 | 19.9 | 0.42 | 27 | 6 | 96.5 | 3.5 | 0.0 | 0.0 | 17.5 | 66.1 | 16.4 | 40.7 | 12.1 | 12.1 | 35.0 | 60.7 | 39.3 | 2 |
| S | 470 | 74.1 | 7.3 | 0.8 | 2.7 | 3.9 | 0.3 | 11.0 | 0.13 | 29 | 5 | 96.6 | 2.9 | 0.4 | 0.2 | 1.3 | 58.6 | 40.1 | 38.6 | 20.9 | 5.3 | 35.2 | 31.7 | 68.3 | 2 |
| S | 308 | 78.8 | 3.1 | 0.7 | 3.8 | 2.3 | 6.3 | 5.0 | 0.10 | 27 | 5 | 97.7 | 0.9 | 0.7 | 0.7 | 1.1 | 84.1 | 14.8 | 63.1 | 19.8 | 2.4 | 14.7 | 36.7 | 63.3 | 2 |
| S | 327 | 76.9 | 7.0 | 0.9 | 1.7 | 2.2 | 0.0 | 11.3 | 0.11 | 31 | 6 | 96.0 | 3.4 | 0.6 | 0.0 | 0.6 | 55.9 | 43.5 | 88.4 | 1.9 | 1.2 | 8.5 | 7.2 | 92.8 | 2 |
| S | 101 | 51.3 | 16.2 | 0.0 | 4.5 | 2.6 | 0.0 | 25.3 | 0.30 | 19 | 5 | 89.9 | 7.6 | 1.3 | 1.3 | 2.5 | 84.8 | 12.7 | 74.0 | 2.7 | 9.6 | 13.7 | 26.0 | 74.0 | 2 |
| S | 234 | 29.6 | 17.1 | 1.8 | 0.9 | 4.9 | 42.7 | 3.0 | 0.38 | 31 | 6 | 92.8 | 3.1 | 1.0 | 3.1 | 39.5 | 44.4 | 16.0 | 34.2 | 2.5 | 13.9 | 49.4 | 64.6 | 35.4 | 3 |
| S | 837 | 71.4 | 11.8 | 0.6 | 5.6 | 1.8 | 0.0 | 9.0 | 0.15 | 18 | 3 | 94.7 | 5.3 | 0.0 | 0.0 | 12.4 | 22.4 | 65.2 | 82.9 | 0.5 | 1.0 | 15.7 | 17.1 | 82.9 | 4 |
| B | 265 | 66.3 | 19.2 | 0.5 | 0.5 | 0.7 | 0.0 | 12.9 | 0.17 | 28 | 5 | 90.5 | 8.8 | 0.4 | 0.4 | 2.1 | 71.4 | 26.6 | 39.0 | 22.8 | 8.7 | 29.5 | 44.4 | 55.6 | 1 |
| B | 375 | 75.3 | 11.6 | 0.9 | 2.0 | 1.0 | 0.3 | 8.8 | 0.12 | 33 | 5 | 90.1 | 8.1 | 1.6 | 0.2 | 7.2 | 84.5 | 8.2 | 43.6 | 19.6 | 21.4 | 15.5 | 62.6 | 37.4 | 1 |
| B | 143 | 70.2 | 7.6 | 3.8 | 0.8 | 1.7 | 0.0 | 16.0 | 0.15 | 26 | 6 | 94.6 | 5.4 | 0.0 | 0.0 | 0.7 | 60.7 | 38.7 | 19.3 | 32.7 | 8.0 | 40.0 | 49.3 | 50.7 | 1 |
| M | 199 | 83.2 | 6.6 | 0.0 | 0.3 | 1.8 | 0.0 | 8.1 | 0.07 | 17 | 5 | 99.6 | 0.4 | 0.0 | 0.0 | 4.2 | 81.9 | 13.9 | 87.3 | 3.8 | 0.0 | 8.9 | 20.4 | 79.6 | 5 |
| M | 503 | 80.4 | 9.1 | 0.7 | 0.8 | 2.0 | 0.0 | 7.0 | 0.09 | 25 | 5 | 97.8 | 2.2 | 0.0 | 0.0 | 0.0 | 90.0 | 10.0 | 97.6 | 0.5 | 0.7 | 1.2 | 8.9 | 91.1 | 5 |
| M | 315 | 68.4 | 8.8 | 0.2 | 4.6 | 0.9 | 11.7 | 5.5 | 0.17 | 31 | 6 | 98.0 | 2.0 | 0.0 | 0.0 | 1.4 | 84.4 | 14.2 | 37.2 | 15.1 | 33.2 | 14.5 | 63.4 | 36.6 | 5 |
| M | 116 | 48.5 | 20.2 | 1.8 | 4.3 | 6.7 | 0.0 | 18.4 | 0.36 | 19 | 5 | 67.1 | 31.6 | 1.3 | 0.0 | 3.0 | 68.2 | 28.8 | 45.5 | 30.3 | 0.0 | 24.2 | 59.1 | 40.9 | 3 |
| P | 1105 | 69.8 | 18.1 | 0.2 | 3.1 | 1.9 | 0.0 | 6.9 | 0.15 | 36 | 6 | 67.8 | 23.5 | 7.6 | 1.1 | 0.6 | 48.2 | 51.1 | 69.7 | 2.1 | 2.9 | 25.2 | 30.7 | 69.3 | 6,7 |
| P | 2321 | 76.8 | 8.7 | 2.5 | 3.1 | 0.6 | 0.3 | 8.0 | 0.11 | 47 | 6 | 64.3 | 30.9 | 3.0 | 1.8 | 1.3 | 67.0 | 31.7 | 68.2 | 7.7 | 3.8 | 20.2 | 29.8 | 70.2 | 7,8 |
| P | 801 | 92.8 | 3.6 | 0.3 | 0.6 | 0.9 | 0.2 | 1.5 | 0.03 | 33 | 5 | 83.7 | 14.5 | 1.6 | 0.2 | 0.3 | 82.5 | 17.3 | 59.2 | 8.3 | 4.3 | 28.2 | 31.2 | 68.8 | 3 |
| P | 126 | 92.9 | 1.0 | 0.3 | 3.8 | 1.3 | 0.0 | 0.6 | 0.03 | 21 | 4 | na | na | na | na | na | na | na | na | na | na | na | na | na | 9 |
| P | 519 | 76.9 | 7.3 | 0.9 | 4.0 | 0.3 | 0.0 | 10.5 | 0.11 | 30 | 6 | 93.9 | 5.5 | 0.6 | 0.0 | 0.0 | 94.9 | 5.1 | 96.9 | 0.3 | 1.0 | 1.8 | 22.3 | 77.7 | 10 |
| P | 182 | 80.1 | 7.2 | 0.3 | 0.7 | 2.9 | 1.6 | 7.2 | 0.09 | 23 | 5 | 93.2 | 6.4 | 0.2 | 0.2 | 0.3 | 51.4 | 48.4 | 27.9 | 17.5 | 4.4 | 50.2 | 33.7 | 66.3 | 10 |

Notes:

Habitat type: R – river, S - small watercourse (stream, canal), B – backwater (or oxbow), M – marsh, P – pond and lake; n – number of spraints analysed; food categories: F – fish, A – amphibians, R – reptiles, B – birds, M – mammals, C – crayfish, I – other aquatic invertebrates;

%RFO – percentage relative frequency of occurrence; B_A_ Levins’ (1968) standardised measure of niche breadth; NPS – number of prey species; NFF – total number of fish families; na – not available in original source; fish weight categories: 1 – <100 g, 2 – 101-500 g, 3 – 501-1000 g, 4 – >1000 g; fish guild categories: R – rheophilic (preferring flowing water), E – eurytopic (tolerant of both flowing and standing waters), S – stagnophilic (preferring stagnant waters); main habitat zone of fish: L – littoral (littoral, or shorezone), M – metaphyton (or aquatic plants, primarily shoreline reed grass, tangle, or under shoreline bush), P – pelagic (occurring in open water areas), B –benthic (or occurring near the bed /in the layer of water directly above the bed/); N – native, A – alien (non-native), sources: 1 – Lanszki and Sallai (2006), 2 – Lanszki et al. (2009), 3 - Lanszki and Molnár (2003), 4 – Lanszki et al. (1999), 5 – Lanszki and Széles (2006), 6 – Lanszki and Körmendi (1996), 7 – Lanszki et al. (2001), 8 – Lanszki et al. (2006), 9 – Nagy 2002, 10 – Lanszki and Széles (2010).
