# Supplementary material for: Diet of otters (Lutra lutra) in various habitat types in the Pannonian biogeographical region compared to other regions of Europe
Source: PeerJ. 2016 Aug 18;4:e2266. doi: 10.7717/peerj.2266 (PMC4994076; doi:10.7717/peerj.2266)
Supplement: Table S2 [file peerj-04-2266-s002.doc]

**Table S2. Diet composition of otters (*Lutra lutra*) in freshwater habitats of different biogeographical regions, in Europe.**

| Bio. | Hab. | n | Food types | | | | | | | B_A_ | Source |
| --- | --- | --- | --- | --- | --- | --- | --- | --- | --- | --- | --- |
| reg. | type |  | F | A | R | B | M | C | I |  |  |
| B | P | 276 | 63.7 | 9.4 | 0.0 | 5.2 | 17.5 | 2.9 | 1.3 | 0.21 | 11 |
| B | R | 137 | 73.4 | 13.1 | 0.0 | 0.7 | 9.7 | 0.6 | 2.6 | 0.13 | 11 |
| B | S | 361 | 60.7 | 18.5 | 0.0 | 4.0 | 12.3 | 1.7 | 2.7 | 0.23 | 11 |
| B | P | 1593 | 90.3 | 3.4 | 0.0 | 1.4 | 2.1 | 0.5 | 2.3 | 0.04 | 12 |
| B | M | 200 | 89.6 | 4.3 |  | 3.2 | 0.0 | 2.2 | 0.7 | 0.04 | 13 |
| B | R | 350 | 93.5 | 1.6 | 0.0 | 3.9 | 0.1 | 0.0 | 0.9 | 0.02 | 14 |
| A | R | 978 | 85.1 | 12.5 | 0.0 | 1.3 | 0.8 | 0.0 | 0.2 | 0.06 | 15 |
| A | S | 253 | 91.8 | 0.6 | 0.0 | 1.1 | 4.2 | 0.0 | 2.3 | 0.03 | 16 |
| A | P | 389 | 91.4 | 0.5 | 0.0 | 7.1 | 1.0 | 0.0 | 0.0 | 0.03 | 16 |
| A | P | 1547 | 92.7 | 0.5 | 0.0 | 4.6 | 1.2 | 0.0 | 1.0 | 0.03 | 17 |
| A | S | 858 | 69.8 | 7.0 | 0.0 | 4.2 | 0.4 | 0.0 | 18.6 | 0.15 | 18 |
| A | R | 675 | 81.7 | 5.4 | 0.0 | 1.6 | 6.8 | 0.0 | 4.5 | 0.08 | 17 |
| A | M | 358 | 93.3 | 0.5 | 0.0 | 5.7 | 0.5 | 0.0 | 0.0 | 0.02 | 19 |
| A | R | 161 | 69.0 | 4.0 | 0.0 | 0.0 | 1.0 | 0.0 | 26.0 | 0.14 | 20 |
| A | R | 1253 | 88.0 | 4.0 | 0.1 | 1.0 | 6.5 | 0.0 | 0.0 | 0.05 | 21 |
| A | M | >100 | 78.6 | 10.6 | 0.0 | 1.4 | 4.9 | 2.1 | 2.1 | 0.10 | 22 |
| A | R | 781 | 66.0 | 13.0 | 0.1 | 5.0 | 1.0 | 0.0 | 16.0 | 0.18 | 23 |
| A | R | >100* | 39.7 | 24.0 | 0.0 | 3.0 | 0.0 | 30.3 | 2.7 | 0.37 | 24 |
| A | P | >100* | 59.0 | 25.0 | 0.0 | 5.0 | 0.0 | 10.5 | 0.5 | 0.23 | 24 |
| A | P | 225 | 80.0 | 16.0 | 0.0 | 2.0 | 0.0 | 0.0 | 2.0 | 0.08 | 25 |
| A | P | 235 | 76.0 | 19.0 | 0.0 | 3.0 | 1.0 | 0.0 | 1.0 | 0.10 | 25 |
| A | R | 130 | 68.0 | 13.0 | 0.0 | 2.0 | 15.0 | 0.0 | 2.0 | 0.16 | 25 |
| A | S | 105 | 75.0 | 22.0 | 0.0 | 1.0 | 2.0 | 0.0 | 0.0 | 0.11 | 25 |
| A | R | 220 | 96.7 | 2.6 | 0.0 | 0.0 | 0.7 | 0.0 | 0.0 | 0.01 | 26 |
| A | P | 1018 | 77.0 | 12.0 | 0.0 | 5.0 | 6.0 | 0.0 | 0.0 | 0.11 | 27 |
| C | P | 6606 | 79.1 | 2.2 | 0.0 | 6.7 | 0.1 | 10.3 | 1.6 | 0.09 | 13 |
| C | P | 399 | 81.2 | 0.2 | 0.0 | 5.6 | 0.2 | 10.5 | 2.3 | 0.08 | 13 |
| C | P | 333 | 81.1 | 2.3 | 0.0 | 8.5 | 0.6 | 6.5 | 1.0 | 0.08 | 13 |
| C | P | 264 | 77.5 | 2.0 | 0.0 | 14.8 | 0.0 | 4.1 | 1.6 | 0.10 | 13 |
| C | P | 251 | 78.9 | 1.8 | 0.0 | 15.2 | 0.0 | 2.0 | 2.1 | 0.09 | 13 |
| C | S | 973 | 85.1 | 1.9 | 0.0 | 7.1 | 0.2 | 5.0 | 0.7 | 0.06 | 13 |
| C | S | 586 | 81.5 | 0.8 | 0.0 | 8.2 | 0.2 | 8.5 | 0.8 | 0.08 | 13 |
| C | S | 4888 | 39.7 | 24.4 | 0.0 | 3.0 | 0.6 | 31.3 | 1.0 | 0.36 | 13 |
| C | M | 115 | 73.5 | 8.1 | 0.0 | 8.1 | 2.8 | 4.7 | 2.8 | 0.13 | 13 |
| C | R | 6390 | 58.9 | 18.9 | 0.0 | 1.2 | 1.6 | 14.5 | 4.9 | 0.24 | 28 |
| C | P | 464 | 74.9 | 6.9 | 0.0 | 1.8 | 1.2 | 13.9 | 1.4 | 0.12 | 28 |
| C | S | 2185 | 48.4 | 40.3 | 0.0 | 1.1 | 2.3 | 1.0 | 6.9 | 0.25 | 28 |
| C | P | 220 | 85.5 | 12.7 | 0.0 | 1.8 | 0.0 | 0.0 | 0.0 | 0.06 | 28 |
| C | P | 127 | 70.1 | 24.4 | 0.0 | 1.6 | 1.5 | 0.0 | 2.4 | 0.14 | 28 |
| C | P | 235 | 97.0 | 1.3 | 0.0 | 0.9 | 0.4 | 0.0 | 0.4 | 0.01 | 28 |
| C | R | 704 | 83.9 | 6.6 | 0.3 | 0.5 | 0.5 | 5.6 | 2.6 | 0.07 | 29 |
| C | P | 596 | 93.0 | 0.6 | 0.0 | 0.9 | 0.9 | 0.0 | 4.6 | 0.03 | 30 |
| C | P | 395 | 88.0 | 1.4 | 0.7 | 2.3 | 1.6 | 0.0 | 6.0 | 0.05 | 30 |
| C | R | 123 | 77.8 | 1.3 | 0.0 | 2.5 | 9.6 | 0.0 | 8.8 | 0.10 | 30 |
| C | R | 349 | 83.9 | 2.9 | 0.0 | 0.6 | 1.7 | 0.0 | 10.9 | 0.07 | 31 |
| C | P | 1099 | 93.7 | 2.6 | 0.0 | 0.2 | 0.3 | 2.2 | 1.0 | 0.02 | 32 |
| C | R | 396 | 45.0 | 46.0 | 0.0 | 2.0 | 0.0 | 0.0 | 7.0 | 0.23 | 33 |
| C | R | 3089 | 68.5 | 12.5 | 0.0 | 1.9 | 0.6 | 14.5 | 2.0 | 0.16 | 34 |
| Al | R | 379 | 63.4 | 14.5 | 0.0 | 0.0 | 0.2 | 1.1 | 20.8 | 0.19 | 35 |
| Al | R | 736 | 80.4 | 14.1 | 0.0 | 0.0 | 0.6 | 4.7 | 0.2 | 0.08 | 34 |
| Al | S | 214 | 57.0 | 40.4 | 0.0 | 0.0 | 2.6 | 0.0 | 0.0 | 0.17 | 34 |
| Al | S | 133 | 72.0 | 22.0 | 0.0 | 1.0 | 4.0 | 0.0 | 1.0 | 0.13 | 36 |
| Al | S | 894 | 49.1 | 20.9 | 0.2 | 0.1 | 0.7 | 3.3 | 25.7 | 0.31 | 37 |
| M | S | 2883 | 44.7 | 14.5 | 2.1 | 1.2 | 0.0 | 24.8 | 12.7 | 0.39 | 38 |
| M | R | 916 | 79.0 | 0.6 | 0.5 | 0.5 | 0.8 | 15.8 | 2.8 | 0.09 | 39 |
| M | P | 412 | 79.7 | 5.0 | 1.0 | 0.2 | 0.5 | 8.8 | 4.8 | 0.09 | 39 |
| M | R | 206 | 82.1 | 11.6 | 1.7 | 0.3 | 0.0 | 0.0 | 4.5 | 0.08 | 40 |
| M | S | 334 | 67.1 | 12.5 | 3.1 | 0.3 | 0.3 | 0.0 | 16.7 | 0.17 | 41 |
| M | P | 264 | 37.9 | 7.1 | 0.3 | 0.3 | 0.7 | 31.5 | 22.1 | 0.39 | 41 |
| M | R | 367 | 69.3 | 0.4 | 0.6 | 0.9 | 3.9 | 24.5 | 0.4 | 0.14 | 42 |
| M | R | 108 | 82.7 | 0.0 | 0.6 | 0.6 | 2.4 | 13.1 | 0.6 | 0.07 | 43 |
| M | P | 307 | 68.9 | 12.2 | 0.0 | 0.0 | 0.0 | 0.0 | 18.9 | 0.15 | 44 |
| M | R | 426 | 60.0 | 5.4 | 3.4 | 0.5 | 0.3 | 5.8 | 24.6 | 0.22 | 45 |
| M | S | 506 | 32.4 | 17.6 | 4.7 | 0.0 | 0.0 | 35.0 | 10.2 | 0.45 | 46 |
| M | R | 1323 | 57.3 | 26.0 | 1.7 | 0.5 | 0.4 | 13.1 | 1.0 | 0.24 | 47 |
| M | R | 838 | 52.5 | 34.8 | 3.6 | 1.1 | 0.3 | 2.4 | 5.3 | 0.25 | 48 |
| M | P | 340 | 82.4 | 1.5 | 0.0 | 4.4 | 9.7 | 0.0 | 2.0 | 0.07 | 49 |
| M | S | 340 | 35.3 | 0.0 | 0.0 | 4.2 | 15.5 | 40.8 | 4.2 | 0.36 | 49 |

Notes

Biogeographical regions (Bio. reg.): B – Boreal, A – Atlantic, C – Continental, Al – Alpine, M – Mediterranean, for other abbreviations see Supplementary material Appendix 1. * two sites together: n= 2349, Sources: 11 – Sulkava (1996), 12 – Baltrunaite (2009), 13 – Erlinge (1967), 14 – Erlinge (1969), 15 – Taaström and Jacobsen (1999), 16 – Chanin (1981), 17 – Wise et al. (1981), 18 – Webb (1975), 19 – Miranda et al. (2008), 20 – Bonesi et al. (2004), 21 – Jenkins and Harper (1980), 22 – Lodé (1989), 23 – McFadden and Fairley (1984), 24 – Kyne et al. (1989), 25 – Weber (1990), 26 – Carss et al. (1990), 27 – Jenkins et al. (1979), 28 – Sidorovich (1997), 29 – Libois (1997), 30 – Roche (1998), 31 – Kortan et al. (2010), 32 – Geidezis (1998), 33 – Jedrzejewska et al. (2001), 34 – Brzezinski et al. (2006), 35 – Harna (1993), 36 – Kozena et al. (1992), 37 – Polednik et al. (2004), 38 – Beja (1996), 39 – Sales-Luís et al. (2007), 40 – Marques et al. (2007), 41 – Adrián and Delibes (1987), 42 – Callejo and Delibes (1987), 43 – Melero et al. (2008), 44 – Delibes et al. (2000), 45 – Morales et al. (2004), 46 – Clavero et al. (2004), 47 – Remonti et al. (2008), 48 – Smiroldo et al. (2009), 49 – Gourvelou et al. (2000).
