# Supplementary material for: Diet of otters (Lutra lutra) in various habitat types in the Pannonian biogeographical region compared to other regions of Europe
Source: PeerJ. 2016 Aug 18;4:e2266. doi: 10.7717/peerj.2266 (PMC4994076; doi:10.7717/peerj.2266)
Supplement: Appendix S1 [file peerj-04-2266-s003.doc]

**Appendix S1. Additional references to Tables S1 and S2.**

Adrián MI, Delibes M. Food habits of the otter (*Lutra lutra*) in two habitats of the Donana National Park, SW Spain. J Zool. 1987; 212: 399–406.

Baltrunaite L. Diet of otters in fish farms in Lithuania. Acta Zool Lit. 2009; 19: 182–187.

Beja PR. An analysis of otter *Lutra lutra* predation on introduced American crayfish *Procambarus clarkii* in Iberian streams. J Appl Ecol. 1996; 33: 1156–1170.

Bonesi L, Chanin P, Macdonald DW. Competition between Eurasian otter *Lutra lutra* and American mink *Mustela vison* probed by niche shift. Oikos. 2004; 106: 19–26.

Brzezinski M, Romanowski J, Kopczynski L, Kurowicka E. Habitat and seasonal variations in diet of otters, *Lutra lutra* in eastern Poland. Folia Zool. 2006; 55: 337–348.

Callejo A, Delibes M. Diet of the otter *Lutra lutra* (Linnaeus, 1758) in the upper reaches of the River Ebro catchment, northern Spain. Miscellan Zool. 1987; 11: 353–362.

Chanin PRF. The diet of the otter and its relations with the feral mink in two areas of Southwest England. Acta Theriol. 1981; 26: 83–95.

Clavero M, Prenda J, Delbes M. Influence of spatial heterogeneity on coastal otter (*Lutra lutra*) prey consumption. Ann Zool Fenn. 2004; 41: 551–561.

Delibes M, Ferreras P, Blázquez CM. Why the Eurasian otter (*Lutra lutra*) leaves a pond? An observational test of some predictions on prey depletion. Rev Ecol-Terre Vie. 2000; 55: 57–65.

Geidesis LC. Diet of otters (*Lutra lutra*) in relation to prey availability in a fish pond area in Germany. IUCN OSG Bulletin. 2002; 19A: 72–76.

Gourvelou E, Papageorgiou N, Neophytou C. Diet of the otter *Lutra lutra* in lake Kerkini and stream Milli-Aggistro, Greece. Acta Theriol. 2000; 45: 35–44.

Harna G. Diet composition of the otter *Lutra lutra* in the Bieszczady Mountains, south-east Poland. Acta Theriol. 1993; 38: 167–174.

Jenkins D, Walker JGK, McCowan D. Analyses of otter (*Lutra lutra*) faeces from Deeside, N.E. Scotland. J Zool. 1979; 187: 235–244.

Jenkins D, Harper RJ. Ecology of otters in northern Scotland II. Analyses of otter (*Lutra lutra*) and mink (*Mustela vison*) faeces from Deeside, N.E. Scotland in 1977-78. J Anim Ecol. 1980; 49: 737–754.

Kortan D, Adámek Z, Vrána P. Otter, *Lutra lutra*, feeding pattern in the Kamenice River (Czech Republic) with newly established Atlantic salmon, *Salmo salar*, population. Folia Zool. 2010; 59: 223–230.

Kozená I, Urban P, Štouračová I, Mazur I. The diet of the otter, *Lutra lutra* Linn. in the Polana protected landscape region. Folia Zool. 1992; 41: 107–122.

Kyne MJ, Smal CM, Fairley JS. The food of otters *Lutra lutra* in the Irish Midlands and a comparison with that of mink *Mustela vison* in the same region. P Roy Irish Acad B. 1989; 89: 33–46.

Lanszki J, Körmendi S. Otter diet in relation to fish availability in a fish pond in Hungary. Acta Theriol. 1996; 41: 127–136.

Lanszki J, Körmendi S, Hancz C, Zalewski A. Feeding habits and trophic niche overlap in a Carnivora community of Hungary. Acta Theriol. 1999; 44: 429–442.

Lanszki J, Széles LG. Diet composition of otters living on abandoned fishpond and gravel pit pond system. Természetvédelmi Közlemények (Nature Conservation Records). 2010; 16: 91–102.

Libois R. Régime et tactique alimentaires de la loutre (*Lutra lutra*) dans le Massif Central. Vie Milieu. 1997; 47: 33–45.

Lodé T. La loutre en Loire-Atlantique *Lutra lutra* L. 1758. Bull Soc nat Ouest de la France. 1989; 11: 69–76.

Marques C, Rosalino LM, Santos-Reis M. Otter predation in a trout fish farm of central-east Portugal: preference for ‘fast-food’. River Res Appl. 2007; 23: 1147–1153.

McFadden YMT, Fairley JS. Food of otters *Lutra lutra* (L.) in an Irish limestone river system with special reference to the crayfish *Austropotamobius pallipes* (Lereboullet). J Life Sci R Dubl S. 1984; 5: 65–76.

Melero Y, Palazón S, Bonesi L, Gosálbez J. Feeding habits of three sympatric mammals in NE Spain: the American mink, the spotted genet, and the Eurasian otter. Acta Theriol. 2008; 53: 263–273.

Miranda R, Copp GH, Williams J, Beyer K, Gozlan RE. Do Eurasian otters *Lutra lutra* (L.) in the Somerset Levels prey preferentially on non-native fish species? Fund Appl Limnol. 2008;172: 339–347.

Morales JJ, Lizana M, Acera F. Ecología trófica de la nutria paleártica *Lutra lutra* en el río Francia (Cuenca del Tajo, Salamanca). Galemys. 2004; 16: 57–77.

Nagy D. Data on the feeding biology of otter (*Lutra lutra* L.) in the lakes Balaton and Kis-Balaton in Hungary. Opusc Zool Budapest. 2002; 34: 59–66

Polednik L, Mitrenga R, Poledniková K, Lojkásek B. The impact of methods of fishery management on the diet of otters (*Lutra lutra*). Folia Zool. 2004; 53: 27–36.

Remonti L, Prigioni C, Balestrieri A, Sgrosso S. Trophic flexibility of the otter (*Lutra lutra)* in southern Italy. Mamm Biol. 2008; 73: 293–302.

Roche K. The diet of otters. In: Dulfer R., Roche K, editors. First phase report of the Trebon otter project. Scientific background and recommendations for conservation and management planning. Nature and environment, no. 93. Strasbourg: Council of Europe Publishing; 1998. pp. 57–71.

Ruiz-Olmo J, Lopez-Martin JM, Palazon S. The influence of fish abundance on the otter (*Lutra lutra*) populations in Iberian Mediterranean habitats. J Zool. 2001; 254: 325–336.

Sales-Luís T, Pedroso NM, Santos-Reis M. Prey availability and diet of the Eurasian otter (*Lutra lutra*) on a large reservoir and associated tributaries. Can J Zool. 2007; 85: 1125–1135.

Sidorovich VE. Mustelids in Belarus. Minsk: Zolotoy Uley Publisher; 1997.

Sulkava RT. Diet of otters *Lutra lutra* in central Finland. Acta Theriol. 1996; 41: 395–408.

Webb JB. Food of the otter (*Lutra lutra*) on the Somerset levels. J Zool. 1975;177: 486–491.

Weber J-M. Seasonal exploitation of amphibians by otters *Lutra lutra* in north-east Scotland. J Zool. 1990; 220: 641–651.
